# Butanol–isopropanol fermentation with oxygen-tolerant *Clostridium beijerinckii *XH29

**DOI:** 10.1186/s13568-022-01399-6

**Published:** 2022-05-14

**Authors:** Xiuqing Yao, Quan Zhang, Yixuan Fan, Xinyang Xu, Ziyong Liu

**Affiliations:** 1 School of Resources and Civil Engineering, Northeastern University, Shenyang, 110819 China; 2 Dalian Research Institute of Petroleum and Petrochemicals, Dalian, 116045 China; 3 Shandong Provincial Key Laboratory of Synthetic Biology, Key Laboratory of Biofuels, Qingdao Institute of Bioenergy and Bioprocess Technology, Chinese Academy of Sciences, No. 189 Songling Road, Qingda, 266101 China; 4 School Environmental & Safety Engineering, Liaoning Petrochemical University, Fushun, 113001 China; 5 University of Chinese Academy of Sciences, Beijing, 100049 China

**Keywords:** Butanol, Isopropanol, Oxygen tolerance, Corn stover hydrolysate, UV mutagenesis

## Abstract

Acetone–butanol–ethanol (ABE) fermentation is a traditional way for solvents production through bioconversion by *Clostridium* species. It is still a challenge to obtain metabolic engineering strains with high ABE yield. Screening strains with remarkable characteristics from nature and improving ABE yield by mutation are viable approaches. *Clostridium beijerinckii* XH 0906, a newly isolated strain, produces butanol and isopropanol (BI) as the main end-products (9.1 g/L BI) during fermentation with glucose as the sole carbon source. The screening process for this strain was performed under aerobic conditions rather than anaerobic environment. Thus, it is a robust stain capable of oxygen-tolerant BI fermentation. Furthermore, *C. beijerinckii* XH 0906 fermented xylose and glucose simultaneously to produce BI. A mutant strain obtained by ultraviolet (UV) mutagenesis, *C. beijerinckii* XH 29, had improved BI production capacity and could produce 17.0 g/L BI and 18.4 g/L BI using glucose or corn stover hydrolysate, respectively as the carbon source. Interestingly, *C. beijerinckii* XH 29 also produced up to 19.3 g/L isopropanol through fermentation of a glucose–acetone mix. These results indicate that *C. beijerinckii* XH 29 is an excellent BI producer with great potential for industrial applications.

**Graphical Abstract:**

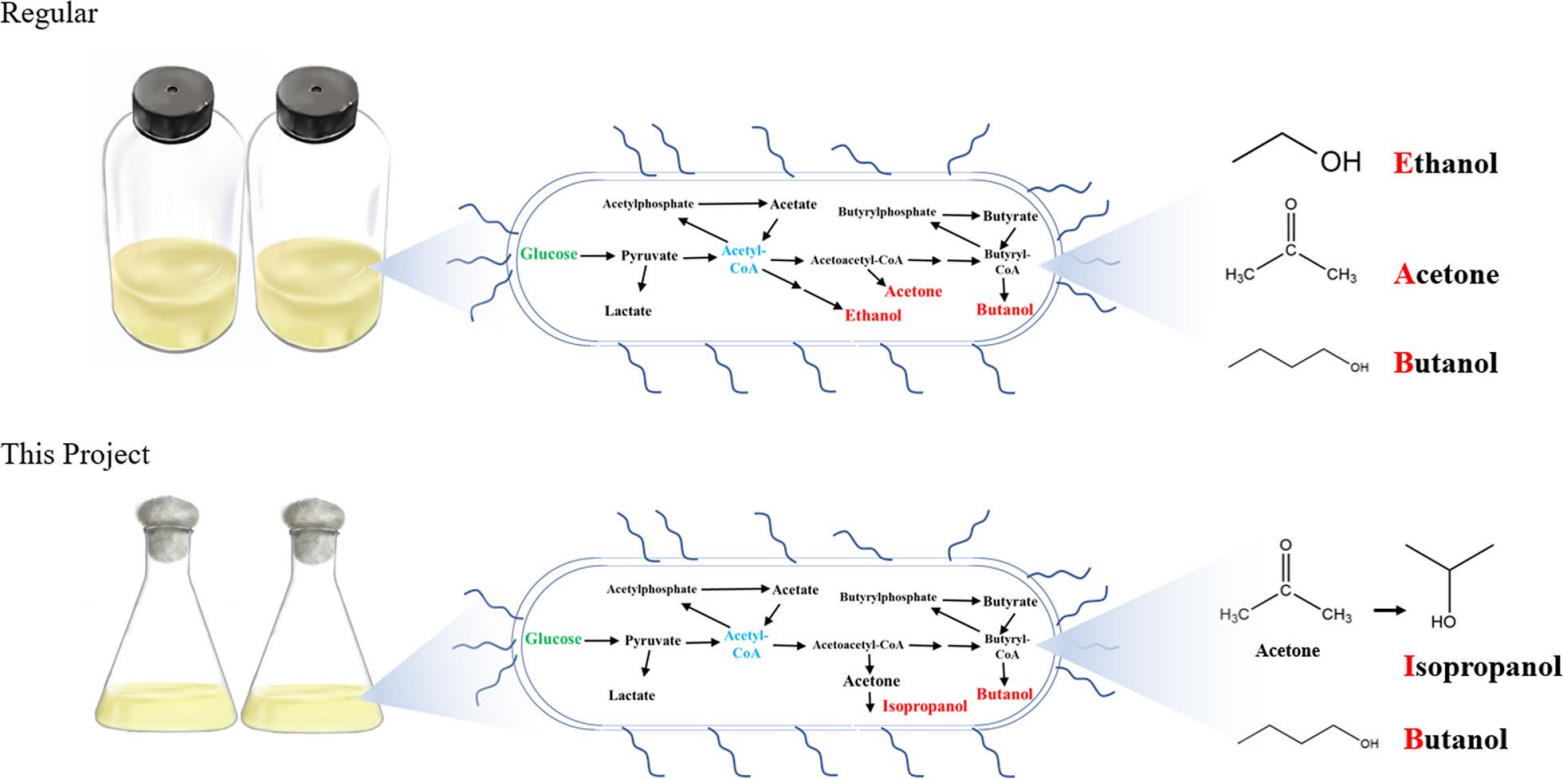

**Supplementary Information:**

The online version contains supplementary material available at 10.1186/s13568-022-01399-6.

## Key points

- A newly isolated strain produces butanol and isopropanol (BI) rather than acetone butanol and ethanol (ABE).
- The strain is oxygen-tolerant and robust in the fermentation.
- A mutant obtained by ultraviolet mutagenesis produces higher levels of BI than the wild type strain using corn stover as a carbon source.

**Supplementary Information:**

The online version contains supplementary material available at 10.1186/s13568-022-01399-6.

## Introduction

Butanol is a chemical material that has wideranging applications in the chemical, pharmaceutical, and petroleum industries (Dürre 1998). Butanol is superior to bioethanol due to its properties of high energy density and low volatility, which makes it an innovative and competitive renewable energy source. Butanol production usually includes chemical synthesis and/or microbial fermentation. Chemical synthesis starts from propylene carbonyl or acetaldehyde, which requires substantial amounts of fossil fuel. However, fossil fuels remain primary energy sources and are nonrenewable, which are likely to result in a future energy shortage in addition to climate change that will choke societal and industrial growth (Moon et al. 2016). Therefore, microbial fermentation is a promising method for future butanol production in the future.

Biobutanol is usually produced by *Clostridium* species. Since the 1900s, *Clostridium acetobutylicum* has been used for butanol and acetone production, with additional butanol-producing microorganisms, such as *C. beijerinckii* and *C. saccharoperbutylacetonicum,* which have been isolated for butanol-producing properties over time. The fermentation process for butanol is biphasic and known as acetone, butanol, and ethanol (ABE) fermentation, which is summarized by several reviews (Lutke-Eversloh and Bahl 2011; Schiel-Bengelsdorf et al. 2013; Zhao et al. 2019). Butanol can also be produced by nonacetone producing *Clostridia*. *C. pasteurianum* can ferment glycerol to produce butanol and 1,3-propanediol (Biebl 2001). *C. carboxidivorans* produces butanol, ethanol, and hexanol by fermenting syngas (Fernandez-Naveira et al. 2016). More recently, engineered strains produce butanol through isopropanol, butanol, and ethanol fermentation (Dai et al. 2012; Dusseaux et al. 2013). The diversity of fermentation products reflects the unique personalities of butanol-producing *Clostridium* species.

Lignocellulosic biomass is an abundant and sustainable resource with great potential as a feedstock for microbial fermentation. Since the beginning of the twenty-first century, many investigations have studied on the use of lignocellulosic hydrolysate as substrate for butanol production (Ezeji et al. 2007; Lee et al. 2016; Li et al. 2018; Qureshi et al. 2010; Yan and He 2017; Zhang et al. 2020). However, compared with glucose fermentation, this substrate presents a series of challenges for microbial growth (Li et al. 2018). Lignocellulose must be pretreated to generate soluble sugars for butanol fermentation, a process that also forms inhibitors (Jonsson and Martin 2016; Yan and He 2017). To release the inhibition of ABE fermentation, several detoxification methods have been developed. The over-liming method is an effective way to reduce the inhibition of ABE fermentation from lignocellulose hydrolysate (Liu et al. 2017). Mutant strains with higher inhibitor tolerances have also been obtained by mutagenesis. The *C. beijerinckii* mutant strain IB4 was screened with low-energy ion implantation and exhibited a high level of inhibitor tolerance (Guo et al. 2012). Furthermore, lignocellulosic hydrolysate is a mixture of pentose and hexose, i.e., xylose and glucose. The simultaneous fermentation of these two sugars is another problem that must be solved for efficient ABE production. An engineered strain of *C. acetobutylicum* was reported to consume glucose and xylose during ABE fermentation (Xiao et al. 2011). However, whether this strain is suitable for fermenting lignocellulosic hydrolysate has not been reported. Some satisfactory results have been obtained by directly converting cellulose to butanol by through fermentation by coculture of metabolically engineered *C. cellulovorans* and *C. beijerinckii* (Bao et al. 2019; Wen et al. 2019, 2017). In brief, the efficient fermentation of unpretreated lignocellulosic hydrolysate by butanol-producing *Clostridium* species remains a challenge.

The present study isolated the novel strain of *C. beijerinckii* XH0906, which produces isopropanol and butanol from a glucose and xylose mixture. The screening and cultivation of this strain were conducted using conventional conical flasks rather than anaerobic bottles, which selected for a strain that can keep anaerobic condition. These results further expand our understanding of butanol-producing *Clostridium* species and provide an important means for future butanol metabolism.

## Materials and methods

### Media for cultivation and fermentation

Reinforced clostridial medium (RCM) was used to isolate butanol-producing strains and P2 medium was used for butanol fermentation. In a 100 mL flask, 30 mL medium supplemented with one carbon source (either glucose, glucose–xylose mix, glucose–acetone, or corn stalk hydrolysate) and 1 g/L yeast extract was sterilized at 115 °C for 15 min. Then, upon cooling to room temperature, 0.3 mL of each filter-sterilized P2 stock (solution was added: (1) Buffer: 50 g/L KH_2_PO_4_; 50 g/L K_2_HPO_4_; 220 g/ L ammonium acetate; Mineral: 20 g/L MgSO_4_•7H_2_O; 1 g/L MnSO_4_•H_2_O; 1 g/L FeSO_4_•7H_2_O; 1 g/L NaCl; and (3) Vitamin: 0.1 g/L para-aminobenzoic acid; 0.1 g/ L thiamin; 0.001 g/L biotin) (Liu et al. 2010; Qureshi and Blaschek 1999). To allow sufficient fermentation, the cultures were incubated at 37 °C for 5 days. Soluble starch was the carbon source in the P2 selective plates.

### Screening and purification of butanol-producing strains

Soil samples from a forest in the suburb of Fushun City (China) were screened on RCM. Each 2 g soil sample was added to 50 mL sterilized RCM with xylose as the sole carbon source in a 100 mL conical flask. The flasks were incubated at 80 °C for 10 min, and then transferred to 37 °C incubator for 5 days. When gas was produced by fermentation, the supernatant products were determined using gas chromatography (GC). Microbial cultures with butanol production were inoculated into fresh RCM with xylose as the carbon source. After several passages in fresh medium, a single colony was obtained through the plate streaking method. Pure cultures were sent to BGI (Dalian, China) for 16S rRNA gene sequencing to identify the bacterial genus. The phylogenetic tree was constructed by comparing the 16S sequence with the most closely related sequences available in the GenBank database based on the neighbor-joining method. The NCBI GenBank Database accession number is MZ254751.

### Ultraviolet mutagenesis and mutant selections

Ultraviolet (UV) mutagenesis was induced with a 25 W UV light. *C. beijerinckii* XH0906 cells grown in P2 medium were harvested in the exponential phase. Cell pellets were resuspended in fresh medium and diluted in 9 mm Petri dishes to an optical density of 0.1 at 600 nm. The cells were then irradiated by UV for 20, 40, 60, 80, 100 or 120 s. The irradiated cells were spread on selective agar plates containing soluble starch to isolate high butanol-producing mutants. When colonies became visible on the plates, a KI-I solution was sprayed on the plates to identify starch fermenters. Colonies with large halos on the selective agar plates were target mutants, whose butanol-production abilities were further tested in P2 medium with glucose as the carbon source. The preparation of the KI-I solution is as follows: 3 g potassium iodide is dissolved in 100 mL H_2_O, and 1 g iodine was then added. This solution must be stored in a brown bottle.

### Corn stover pretreatment and hydrolysis

Chipped corn stover was obtained from Jilin, China and pretreated by steam explosion. Steam-exploded corn stover (SECS) was digested with 120 filter paper units [FPU]/mL by Cellic® Ctec 2 (Novozymes, Bagsvaerd, Denmark) and detoxified by over-liming. The mixture was centrifuged, and the supernatant retained as detoxified hydrolysate (DTH). The detailed operation process of DTH was described previously (Liu et al. 2017).

### Analytical methods

Cell growth was determined by measuring the absorbance at 600 nm with a quartz cuvette. A GC/MS system (Agilent 7890A, with a 5975 C mass selective detector) was used to identify isopropanol and butanol. Samples for BI measurement were extracted with a threefold volume of ethyl acetate and separated on a DB-5 ms column (Agilent, USA). Helium was used as the carrier gas. The concentration of glucose, xylose and cellobiose in the medium were determined by the Agilent 1100 high-performance liquid chromatography (HPLC) system with an Aminex HPX-87H column (BioRad Laboratories, USA) equipped with a refractive index detector operated at 35 °C. The column temperature was maintained at 65 °C and 5 mM H_2_SO_4_ was used as the mobile phase at a flow rate of 0.5 mL/min. Acetate and butyrate were also determined by HPLC with an Agilent Hi-Plex H column (Agilent Technologies, Santa Clara, CA, USA). Acetone, isopropanol, and butanol were measured by a GC system (Agilent 7890B, USA) equipped with a flame ionization detector and HP-INNOWax column (30 m length, 0.32 mm inner diameter). Samples for acetone, isopropanol, and butanol measurement were extracted with a threefold volume of ethyl acetate containing 5 g/L isobutanol as an internal standard. The organic phase was taken out for GC analysis. High-purity nitrogen was used as the carrier gas.

## Results

### Isolation, identification, and product analysis of *C. beijerinckii* XH0906

In order to screen oxygen-tolerant butanol-producing strains, the soil samples were cultured in conical flasks. Several bubbles were produced in some flasks the after 3 days of growth. GC was used to analyze the products in the culture supernatants. Butanol was detected in the supernatant, which indicated that some strains could produce solvents in this culture. The microbial strains in the culture were purified by serial dilution on agar plates with xylose as the carbon source. Single colonies were picked and grown in fresh medium and the presence of butanol in the resultant supernatant was determined by GC.

**Fig. 1:**
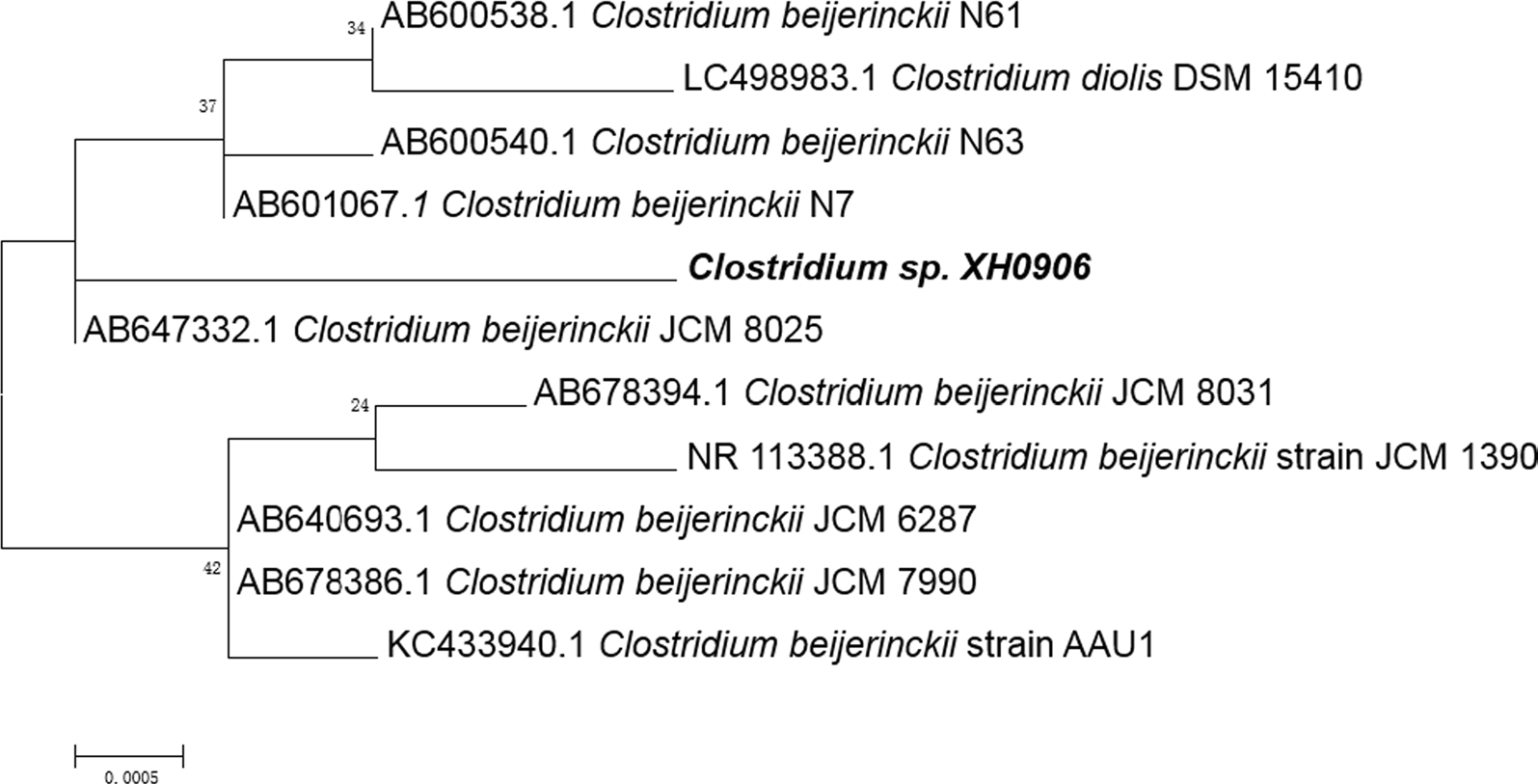
Phylogenetic tree of *Clostridium beijerinckii* XH0906 and closely related strains according to 16S rRNA gene sequences

A pure butanol-producing strain was isolated from soil samples. The solvents in the culture supernatant were determined to be BI by GC–MS (Additional file 1: Fig. S1). The butanol-producing strain was a spore-forming, Gram-positive with rod-shaped cell morphology, and its 16S rRNA gene sequence had a 99.9% sequence similarity to *C. beijerinckii* NCIMB 8052 (Fig. 1). The complete 16S rRNA gene sequence was submitted to the NCBI GenBank Database (accession number MZ254751). The species was confirmed with a carbohydrate metabolism analysis with various substrates using the API 50 CH system (API system, France). Our results confirmed the strain’s identity, which we named *C. beijerinckii* XH0906; it is stored at the China General Microbiological Culture Collection Center (accession number CGMCC No. 9124).

### *C. beijerinckii *XH0906 batch fermentation

**Fig. 2:**
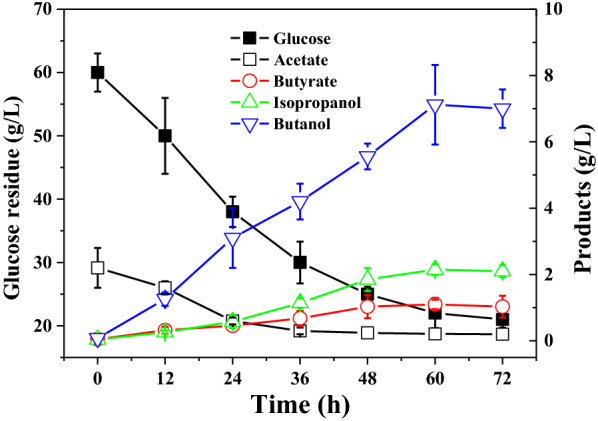
The glucose consumption and product formation during *Clostridium beijerinckii* XH0906 batch fermentation

*Clostridium beijerinckii* XH0906 was cultured in P2 medium to evaluate BI fermentation with glucose as the sole carbon source. This strain produced 7.1 g/L butanol and 2.1 g/L isopropanol (Fig. 2). A small amount of acetate and butyrate (1.0 g/L) were also present in the broth, while ethanol and acetone were not detected. An estimated 39 g/L glucose was consumed by *C. beijerinckii* XH0906 fermentation (Fig. 2).

**Fig. 3:**
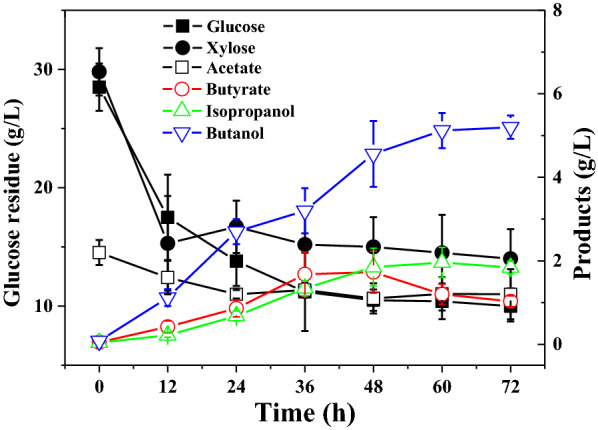
BI fermentation by *C. beijerinckii* XH0906 in P2 medium with glucose and xylose

To approximate the carbon source in pretreated lignocellulosic hydrolysate, *C. beijerinckii* XH0906 was also cultured in P2 medium supplemented with 30 g/L glucose and 30 g/L xylose as the carbon source (Fig. 3). HPLC and GC measurements showed that glucose and xylose were consumed simultaneously during the fermentation and butanol (5.1 g/L), isopropanol (1.9 g/L), and butyrate (1.0 g/L) were detected as end-products (Fig. 3). These results demonstrated the BI-producing ability of *C. beijerinckii* XH0906 metabolizing soluble sugars. However, its BI yield and titer are relatively low, compared with other reported BI-producing strains.

### UV mutagenesis and mutant fermentation

**Fig. 4:**
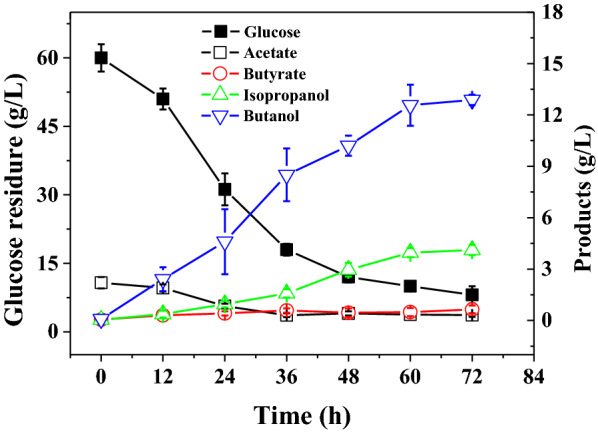
BI fermentation by *C. beijerinckii* XH29 in P2 medium supplemented with glucose

To improve BI titer, UV light was used as a mutagen of *C. beijerinckii* XH0906. Exponential growth-phase cells were UV irradiated for 20, 40, 60, 80, 100, or 120 s, then serially diluted. Colony counting was used to generate a cell survival rate curve by dividing the number of colonies without UV irradiation by the number of colonies that survived UV irradiation (Additional file 1: Fig. S2). Based on the survival curve, we chose 100 s as the irradiation time for the mutant selection. The irradiated cells were spread on selective soluble starch as the carbon source. When colonies were clearly visible after 24 h incubation, the KI-I solution was sprayed on the plates to illuminate starch-absent halos around each colony (Additional file 1: Fig. S3). Colonies with large starch-free circles showed a high level of starch metabolism and suggested higher levels of butanol synthesis. These colonies were selected as target strains for another round of UV mutagenesis. A series of mutants were obtained after 10 rounds of mutagenesis, one of which was the mutant *C. beijerinckii* XH29, which exhibited a remarkable ability for starch metabolism. Batch fermentation of *C. beijerinckii* XH29 in P2 medium with glucose as the carbon source showed that the mutant consumed 52 g/L glucose, which was 33% higher than that of the wild type (Fig. 4). The butanol (12.9 g/L) and isopropanol (4.1 g/L) titers were also significantly higher than the wild type (Figs. 3 and 4). These suggested that we obtained a mutant capable of efficient BI fermentation.

### *C. beijerinckii *XH29 BI fermentation on DTH

**Fig. 5:**
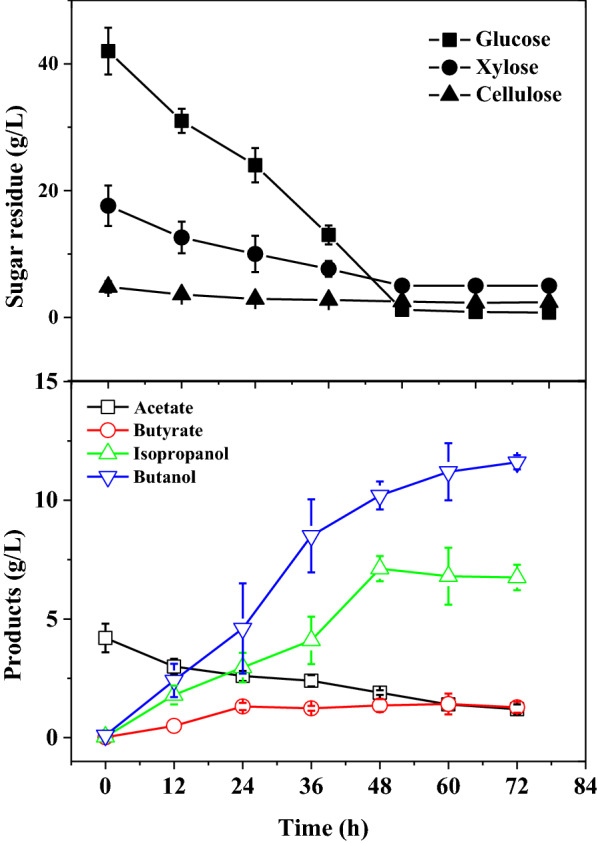
BI fermentation by *Clostridium beijerinckii* XH29 in P2 medium supplemented with DTH

To investigate the fermentation profiles of *C. beijerinckii* XH29 on lignocellulosic hydrolysate, it was cultured in P2 medium with DTH as the carbon source (Fig. 5). DTH contains 64.4 g/L fermentable sugars including 42 g/L glucose, 17.6 g/L xylose, and 4.8 g/L cellobiose. The hydrolysate also contains acetate, formic acid, and other small molecular substances (Additional file 1: Table S1). Results of DTH fermentation showed this mutant consumed 57 g/L soluble sugar indicating that glucose was almost used up in the fermentation. Isopropanol (6.8 g/L) and butanol (11.6 g/L) were detected among the end-products. Compared to glucose fermentation, the isopropanol titer increased by 65% (Figs. 4 and 5). The isopropanol to butanol ratio increased from 0.32 to 0.61, which suggested that DTH doubles the efficiency of butanol fermentation.

### *C. beijerinckii* XH29 BI fermentation of glucose–acetone

**Fig. 6:**
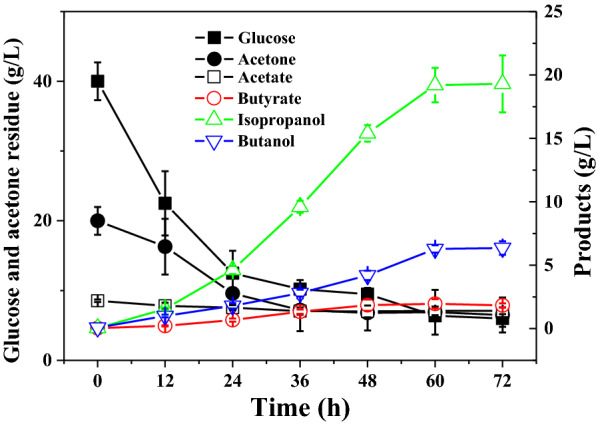
BI fermentation by *Clostridium beijerinckii* XH29 in P2 medium supplemented with glucose and acetone

It is reported that acetone can be convert to isopropanol by one step of reduction reaction. To verify that isopropanol was formed by acetone reduction, *C. beijerinckii* XH29 was cultured in P2 medium with 40 g/L glucose and 20 g/L acetone as the carbon source. Altogether, 36 g/L glucose and 13.5 g/L acetone were consumed during the fermentation process, and 6.4 g/L butanol and 19.3 g/L isopropanol were produced (Fig. 6). These data confirmed that acetone could be used by *C. beijerinckii* XH29 metabolic pathway to synthesize isopropanol via a reduction reaction. Interestingly, butyrate was not detected, which suggested that almost all the carbon sources were converted to BI.

Comparing the cell growth and sugar consumption of the different study conditions, all the fermentations peaked at 24 h (Additional file 1: Fig. S5). The cell density changed along with glucose consumption. These results suggested that the accumulation of *C. beijerinckii* cell biomass was closely related to sugar consumption. Although an increase of isopropanol concentration was detected by the mutant, the cell biomass was not higher than that of the wild type, which further confirmed that acetone was converted to isopropanol during the fermentation of *C. beijerinckii* XH29 (Fig. 6).

## Discussion

### Isolation and metabolic modulation of butanol-producing strains

The most significant characteristic of butanol-producing strain is the production of metabolites that significantly vary by metabolic regulation and fermentation control. *C. acetobutylicum* mainly produces acetone, ethanol and butanol during solventogenesis by pH control during the continuous fermentation (Grimmler et al. 2011). For *Clostridium* sp. A1424, it mainly produces butanol and 1,3-propanediol in the glycerol-glucose batch fermentation (Fernandez-Naveira et al. 2016). Moreover, genetic modification can make *C. acetobutylicum* a nonacetone-producing strain or IBE (isopropanol, butanol, and ethanol)-producing strain (Dai et al. 2012; Dusseaux et al. 2013; Jiang et al. 2009). Compared to traditional ABE fermentation by *Clostridium* species, the main products of *C. beijerinckii* XH0906 were BI. This indicated that *C. beijerinckii* XH0906 has distinct metabolic pathways and redox balance metabolism from ABE-producing strains. Recently, some natural BI producers were reported, including *Clostridium* sp. A1424, *C. beijerinckii* BGS1, and *C. beijerinckii* C-01 (Dalal et al. 2019; Fernandez-Naveira et al. 2016; Zhang et al. 2018). The abundance of nonengineered BI producers suggests that BI fermentation is also an important metabolic choice for bacteria during butanol synthesis. However, no homo-butanol-producing strains, which only produce butanol without isopropanol, acetone or ethanol, have yet been reported (Lutke-Eversloh and Bahl 2011). These examples indicate a flexible and unknown regulation metabolic in butanol-producing *Clostridium* species. To obtain a more efficient homo-butanol-producing strain, much work remains to clarify this mechanism.

Since the batch fermentation was performed in conical flasks, we selected strains like *C. beijerinckii* XH0906 that exhibited oxygen tolerance, which is a great advantage for future industrial production. A significant difference between anaerobic and aerobic bacteria is the energy metabolic mechanism during conversion of pyruvate to acetyl-CoA (Additional file 1: Fig. S4). In anaerobic *Clostridium* species, reduced ferredoxin (Fd_red_) will be formed in the process of catalysis from pyruvate to acetyl-CoA by pyruvate:ferredoxin oxidoreductase, and the released Fd_red_ will synthesize gas hydrogen by coupling hydrogenase to realize the redox balance in the metabolism. Hydrogenase is strictly anaerobic. We speculate that *C. beijerinckii* XH0906 happens to have an oxygen tolerant hydrogenase, which makes it grow and ferment normally in conical flask. Recently, an oxygen tolerant hydrogenase was found and its function was analyzed in *C. beijerinckii* SM10, which provides evidence for us to study the oxygen tolerance mechanism of *C. beijerinckii* XH0906 (Morra et al. 2014, 2016; Winkler et al. 2021). Furthermore, the glucose–xylose fermentation results indicate that *C. beijerinckii* XH0906 is not under carbon catabolite repression (CCR), a universal phenomenon in *Clostridium* species during glucose–xylose fermentation (Mitchell 1998; Ren et al. 2010; Xiao et al. 2011). Based on these characteristics, *C. beijerinckii* XH0906 has incomparable advantages in the fermentation of sugar from enzymatic lignocellulose, in which glucose and xylose are the main components. However, batch fermentation of *C. beijerinckii* XH0906 resulted in less sugar consumption and butanol production than model ABE strains (Jiang et al. 2009; Qureshi et al. 2007).

### Improvement of butanol-producing strains in different conditions

The isolated, natural butanol-producing strains normally need their fermentation titer and yield improved. *C. beijerinckii* BA101 generated by chemical mutagenesis is a remarkable butanol-producer, which produce 33 g/L butanol during batch fermentation (Annous and Blaschek 1991; Chen and Blaschek 1999). *C. beijerinckii* IB4 generated by low-energy ion implantation, is another mutant with higher tolerance to lignocellulose hydrolysate inhibitors. Meanwhile, it produces more butanol than wild type (Guo et al. 2012). In this study, we generated a mutant through UV mutagenesis, indicating that this is an alternative method for isolating efficient butanol fermentation strains.

Numerous studies have reported that the ratio of acetone to butanol is higher following the fermentation of lignocellulosic enzymatic hydrolysate than glucose (Ezeji et al. 2007; Liu et al. 2017; Qureshi et al. 2007; Zhang et al. 2012). Scientists speculate that the soluble lignin generated by the pretreatment process contains active oxidation groups, which inhibit the normal growth and metabolism of butanol-producing *Clostridium* species. To eliminate oxidation stress, the bacteria must consume an equivalent amount of the reducing NAD(P)H. Butanol biosynthesis requires NADH, while acetone biosynthesis from the common precursor acetyl-CoA does not (Additional file 1: Fig. S4). The ratio of acetone to butanol changes according to the supply of NADH, which causes the ratio of acetone to butanol to increase during lignocellulosic enzymatic hydrolysate fermentation. Conversely, the ratio decreases during glucose–glycerol fermentation, as the glycerol metabolic process release more NADH than glucose does (Girbal and Soucaille 1994). In the present study, isopropanol was synthesized from acetone by a one-step reduction reaction (Additional file 1: Fig. S4), which suggests that the increased ratio of isopropanol to butanol was caused for the same reason.

Altogether, this study resulted in the isolation of an oxygen-tolerant BI fermentation strain, *C. beijerinckii* XH0906. Its mutant, *C. beijerinckii* XH29, produced 11.2 g/L butanol and 6.8 g/L isopropanol with DTH as the carbon source. It reached isopropanol production up to 19.3 g/L during glucoseacetone fermentation, which is higher than any previous reports by BI-producing *Clostridium* species (Table 1) (Chen 1995; Chen and Hiu 1986; George et al. 1983; Matsumura et al. 1992; Vrije et al. 2013; Moon et al. 2015; Vieira et al. 2020; Dalal et al. 2019; Zhang et al. 2018). Furthermore, this study described a method for oxygen-tolerant strain isolation and mutation for high level of BI production.

**Table 1:** Comparison of various BI-producing strains

References	Substrate	Srains	Isopropanol (g/L)	Butanol (g/L)
George et al	Glucose 20 g/L	*C. beijerinckii* 2968	0.59	3.32
Chen et al	Glucose 60 g/L	*C. beijerinckii* B-593	0.48	4.57
Chen and Hiu et al	Glucose 60 g/L	*C. beijerinckii* McClung 3081	1.56	6.00
Matsumura et al	Cane molasses 50 g/L	*C. isopropylicum* IAM 19239	4.60	8.30
Vrije et al	Glucose 40 g/L, xylose 20 g/L	*C. beijerinckii* NRRL B593	3.20	6.90
Vieira et al	Glucose 40 g/L	*C. beijerinckii* DSM 6432	1.7	6.5
Moon et al	Glucose 30 g/L	*Clostridium* sp. A1424	4.49	9.43
Dalal et al	Glucose 30 g/L	*C. beijerinckii* C-01	2.5	7.5
Zhang et al	Glucose 60 g/L	*C. beijerinckii* BGS1	3.41	10.21
Zhang et al	Sucrose 60 g/L	*C. beijerinckii* BGS1	2.51	9.79
This study	Glucose 40 g/L, acetone 20 g/L	*C. beijerinckii* XH29	6.8	11.6
This study	DTH, glucose 42 g/L, xylose 17.6 g/L, 4.8 g/L cellobiose	*C. beijerinckii* XH29	19.3	6.4

## Supplementary Information

**Additional file 1:Figure S1** The determination of butanol and isopropanol by GS-MS; **Figure S2** The survival rate curve of UV irradiated; **Figure S3** The selective plate of mutants; **Figure S4** The schematic metabolic pathways in butanol producing clostridia; **Figure S5** Cell growth and sugar consumption under different conditions in this study; **Table S1** Compositional analysis of SECS hydrolysates.

## Data Availability

All data generated or analyzed during this study are included in this published article and its supplementary information file. The complete sequence of 16S rRNA gene was submitted to the NCBI GenBank Database. The accession number in NCBI GenBank Database is MZ254751.
