# Supplementary material for: Butanol–isopropanol fermentation with oxygen-tolerant Clostridium beijerinckii XH29
Source: AMB Express. 2022 May 14;12:57. doi: 10.1186/s13568-022-01399-6 (PMC9107568; doi:10.1186/s13568-022-01399-6)
Supplement: Supplementary file 1 — Additional file 1:Figure S1 The determination of butanol and isopropanol by GS-MS; Figure S2 The survival rate curve of UV irradiated; Figure S3 The selective plate of mutants; Figure S4 The schematic metabolic pathways in butanol producing clostridia; Figure S5 Cell growth and sugar consumption under different conditions in this study; Table S1 Compositional analysis of SECS hydrolysates. [file 13568_2022_1399_MOESM1_ESM.pdf]

AMB Express

Supplemental Materials for

Butanol-isopropanol Fermentation Using a Robust Oxygen-tolerant *Clostridium beijerinckii* XH29

Xiuqing Yao[1,4,#], Quan Zhang[2,#], Yixuan Fan[3,5], Xinyang Xu[1,*], Ziyong Liu[3,*]

[1]College of Resources and Civil Engineering, Northeastern University, Shenyang 110819, China; [2]Dalian Research Institute of Petroleum and Petrochemicals, Dalian 116045, China; [3]Shandong Provincial Key Laboratory of Synthetic Biology, Key Laboratory of Biofuels, Qingdao Institute of Bioenergy and Bioprocess Technology, Chinese Academy of Sciences, Qingdao 266101, China; [4]School Environmental &Safety Engineering, Liaoning Petrochemical University, Fushun 113001, China; [5]University of Chinese Academy of Sciences, Beijing 100049, China.

*Corresponding authors: Qingdao Institute of Bioenergy and Bioprocess Technology, Chinese Academy of Sciences, No. 189 Songling Road, Qingdao 266101, China. Tel.: +86-532-80662656; Fax: +86-532-80662778. E-mail: liuzy@qibebt.ac.cn (Z. Liu); School of Resources and Civil Engineering, Northeastern University, Shenyang 110819, China. Tel.: +86-24-83672113; E-mail: xuxinyang@mail.neu.edu.cn (X. Xu)

[#]Both authors contributed equally to this work.

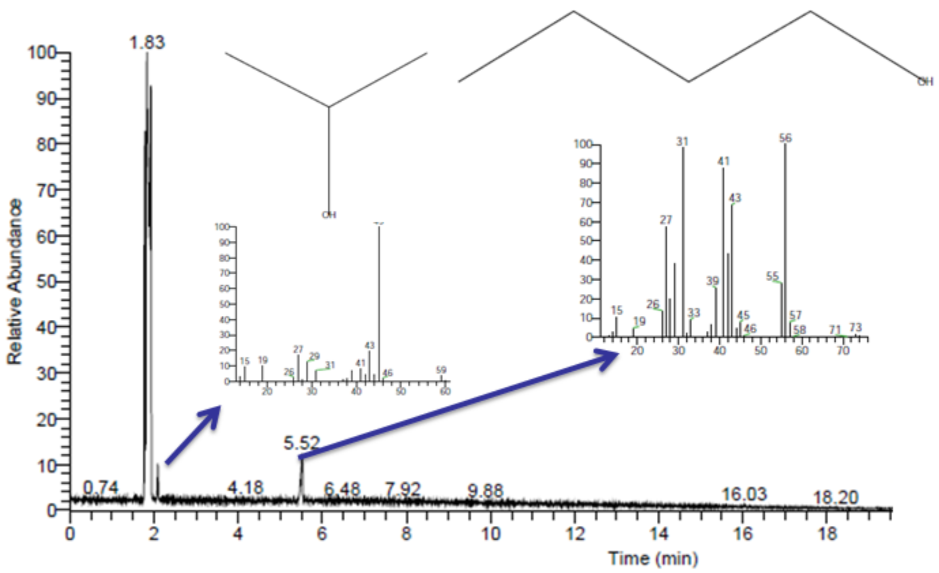

Fig. S1 The determination of butanol and isopropanol by GS-MS

Figure S2 The survival rate curve of UV irradiated

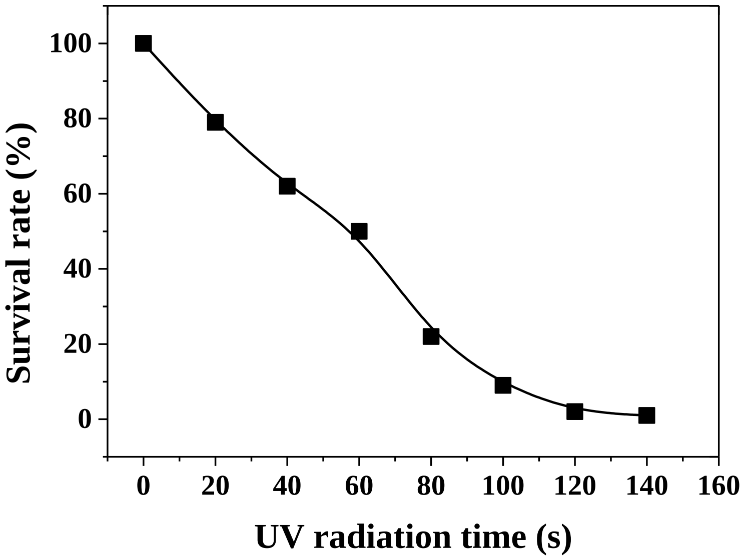

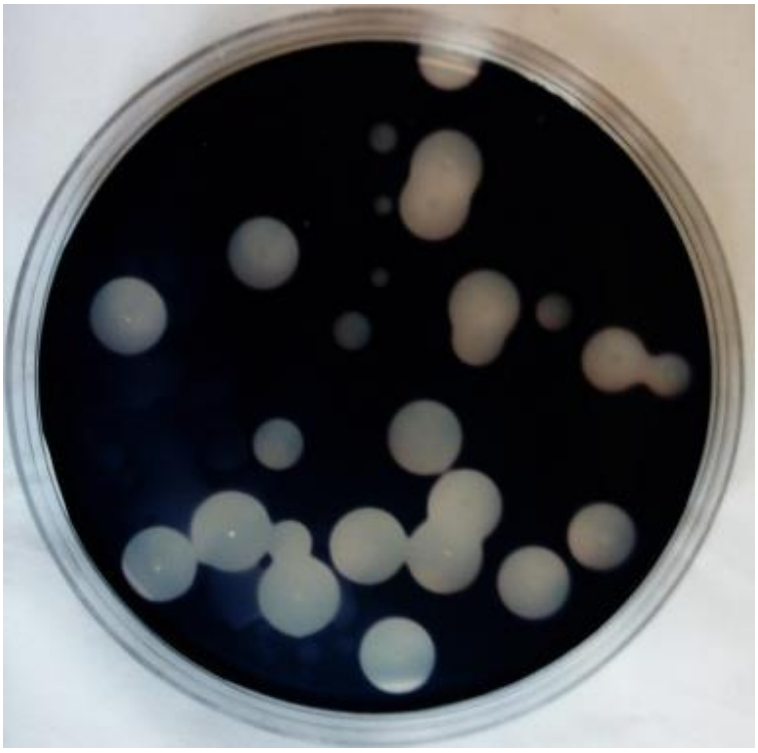

Figure S3 The selective plate of mutants

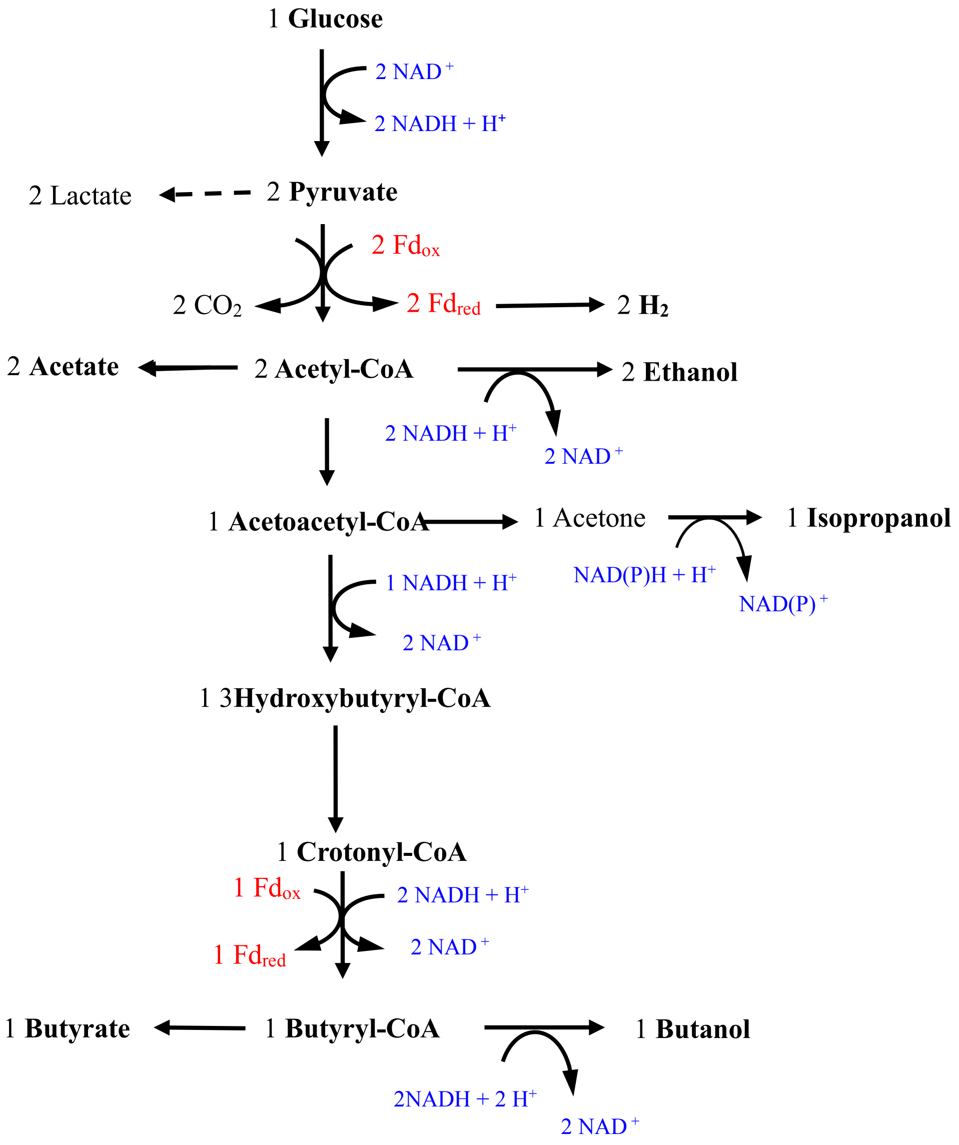

Figure S4 The schematic metabolic pathways in butanol producing clostridia

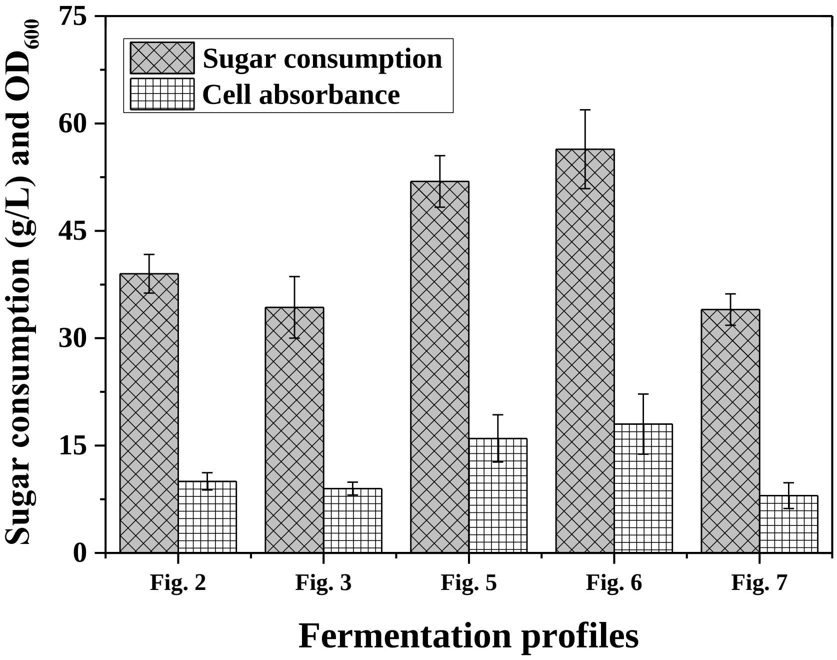

Figure S5 Cell growth and sugar consumption under different conditions in this study

Table S1 Compositional analysis of SECS hydrolysates

Sugars	Conc. (g/L)	Others	Conc. (mg/L)
	DTH[b]		DTH[b]
Glucose	42.1± 3.7	Furfural	200 ± 13
Xylose	17.6 ± 1.2	HMF	120 ± 40
Cellobiose	4.8 ± 0.9	Formic acid	540 ± 100
Total[a]	64.5	Acetate	2000 ± 600

[a]Total indicates the total sugars of glucose, cellulose and xylose determined by HPLC. [b]DTH indicates detoxification hydrolysate.
